# Mechanism and Predictive Role of NUB1 Protein in Oestrogen Receptor Pathway of FEC-Treated Breast Cancer Patients

**DOI:** 10.3390/biomedicines13061307

**Published:** 2025-05-27

**Authors:** Maria Arshad, Amira Raudhah Abdullah, Fuad Ismail, Francesco Pezzella, Azyani Yahaya, Geok-Chin Tan, Suet Lin Chia, Md Salzihan Md Salleh, Noraidatulakma Abdullah, Ka-Liong Tan

**Affiliations:** 1 Faculty of Medicine & Health Sciences, Universiti Sains Islam Malaysia (USIM), Persiaran Ilmu, Nilai 71800, Malaysia; maria@raudah.usim.edu.my (M.A.); amiraraudhah@usim.edu.my (A.R.A.); 2 Department of Radiotherapy & Oncology, Universiti Kebangsaan Malaysia Medical Centre, Jalan Yaacob Latif, Bandar Tun Razak, Kuala Lumpur 56000, Malaysia; fuad2305@yahoo.com; 3 Tumour Pathology Laboratory, Nuffield Division of Clinical Laboratory Sciences, Radcliffe Department of Medicine, John Radcliffe Hospital, Headington, Oxford OX3 9DU, UK; francesco.pezzella@ndcls.ox.ac.uk; 4 Department of Diagnostic and Laboratory Service, Hospital Canselor Tuanku Muhriz, Universiti Kebangsaan Malaysia Medical Centre, Jalan Yaacob Latif, Bandar Tun Razak, Kuala Lumpur 56000, Malaysia; azyani@hctm.ukm.edu.my; 5 Department of Pathology, Faculty of Medicine, Universiti Kebangsaan Malaysia, Jalan Yaacob Latif, Bandar Tun Razak, Kuala Lumpur 56000, Malaysia; tangc@hctm.ukm.edu.my; 6 UPM-MAKNA Cancer Research Laboratory, Institute of Bioscience, Universiti Putra Malaysia, Serdang 43400, Malaysia; suetlin@upm.edu.my; 7 Department of Microbiology, Faculty of Biotechnology and Biomolecular Sciences, Universiti Putra Malaysia, Serdang 43400, Malaysia; 8 Department of Pathology, School of Medical Sciences, Universiti Sains Malaysia, Kubang Kerian 16150, Malaysia; salzihan@usm.my; 9 UKM Medical Molecular Biology Institute, Universiti Kebangsaan Malaysia, Jalan Yaacob Latif, Bandar Tun Razak, Kuala Lumpur 56000, Malaysia; noraidatulakma@ukm.edu.my

**Keywords:** breast cancer, NUB1, ERα, FEC treatment, cell cycle arrest

## Abstract

**Introduction:** NEDD8 Ultimate Buster 1 (NUB1) is a regulator of the cell cycle and a prognostic marker in cancer patients. However, its role in breast cancer (BC) and its response to 5-fluorouracil, epirubicin, and cyclophosphamide (FEC) treatment remain unclear. This study investigated NUB1’s predictive value in FEC treatment and its mechanistic interaction with the oestrogen receptor (ER) in BC. **Methods:** MDA-MB-231 and MCF-7 cells were treated with FEC and analysed via flow cytometry for cell cycle distribution. Western blotting assessed NUB1 and ERα expression, while immunohistochemistry was conducted on a retrospective cohort (*n* = 85) from Malaysian hospitals to evaluate the clinical significance of NUB1 expression. **Results:** FEC treatment induced S and G2 phase cell cycle arrest in MDA-MB-231 cells (*p* = 0.04 and *p* = 0.02, respectively), accompanied by NUB1 upregulation. In MCF-7 cells, G2/M arrest was observed (*p* = 0.01), with reduced ERα expression and increased NUB1 levels in both cell lines. Lower cytoplasmic NUB1 expression was associated with poorer overall survival (OS) (HR = 0.60; 95% CI = 0.32–1.11; *p* = 0.10). Patients with low NUB1 and low ER expression showed the worst OS outcomes. **Discussion:** NUB1 upregulation following FEC treatment led to cell cycle arrest in ER-negative cells, whereas ERα suppression failed to induce S-phase arrest in ER-positive cells. Low NUB1 expression predicted poorer OS and increased BC recurrence. **Conclusions:** By integrating in vitro and clinical data, this study suggests that NUB1 may serve as a predictive biomarker in FEC-treated breast cancer. Larger studies are needed to validate and establish NUB1’s predictive role in FEC-treated patients.

## 1. Introduction

Breast cancer (BC) remains a leading cause of cancer-related mortality worldwide, with significant epidemiological variability across regions. In Malaysia, BC incidence is rising disproportionately among younger women, with 13% of cases diagnosed before the age of 40, compared to 4.4% in the United States [1,2,3,4]. The higher incidence of BC among Malaysian women under 40, compared to that in the U.S., is attributed to genetic predispositions (e.g., higher BRCA1/2 mutation rates), aggressive tumour subtypes (e.g., triple-negative BC), and lifestyle factors (e.g., diet, delayed childbirth, obesity), compounded by late-stage diagnoses linked to cultural healthcare practices [5]. Globally, BC accounted for 2.3 million cases and 685,000 deaths in 2020 [3], underscoring the urgency for improved therapeutic strategies. Current clinical management relies on biomarkers such as oestrogen receptor (ER), progesterone receptor (PR), and HER2 to guide treatment [6,7,8]. However, suboptimal treatment allocation, resistance to conventional FEC chemotherapy (5-fluorouracil, epirubicin, and cyclophosphamide), and its associated toxicities (e.g., cardiotoxicity and cognitive impairment) [9,10,11] highlight an unmet need for predictive biomarkers to optimise patient outcomes and personalise FEC-based regimens.

FEC chemotherapy, a common therapeutic approach for BC, involves administering a combination of drugs [12]. The Fédération Nationale des Centres de Lutte Contre le Cancer (FNCLCC) PACS 01 Trial recommends standard doses of 5-FU (500–600 mg/m^2^), epirubicin (75–90 mg/m^2^), and cyclophosphamide (500 mg/m^2^) for patients with node-positive early BC who have undergone primary surgery for unilateral operable carcinoma of the breast (stage < T4a). These drugs are administered in cycles, with a 21-day interval between cycles [13]. Adjuvant FEC chemotherapy lasting 4–6 cycles as outpatient treatment is associated with common side effects such as nausea, vomiting, hair loss, fatigue, and reduced immunity, and some rare effects such as impaired cognitive function, neutropenia, fertility issues, and cardiotoxicities [10,14,15]. FEC exerts its anti-cancer effects through distinct mechanisms: 5-FU induces S-phase arrest via thymidylate synthase inhibition [16], epirubicin triggers G2 arrest via topoisomerase II inhibition [17], and cyclophosphamide causes DNA crosslinking [18]. While effective, interpatient variability in FEC response remains poorly understood.

Emerging evidence implicates ubiquitin-like proteins in chemoresistance [19], notably neural precursor cell-expressed developmentally downregulated protein 8 (NEDD8), which regulates cell cycle proteins such as cyclin E and p27 through SCF ubiquitin ligases [20,21]. NEDD8 Ultimate Buster 1 (NUB1), an interferon-inducible protein, degrades NEDD8 conjugates and modulates cell cycle progression [22,23]. NUB1 controls cell cycle progression by recruiting NEDD8 for degradation and negatively regulating the NEDD8 conjugation system [20]. NUB1 depletion arrests BC cells in the G0/G1 phase and influences the activity of p27^Kip1^, a cyclin-dependent kinase (CDK) inhibitor [24]. Elevated NUB1 levels suppress tumour growth in gastric cancer by promoting p27^Kip1^ degradation, allowing the cell cycle to continue [24,25]. In gastric cancer, NUB1 suppresses tumour growth by promoting p27^Kip1^ degradation [24], yet its role in BC, particularly in ERα signalling and FEC response, remains unexplored.

This study hypothesised that FEC chemotherapy upregulates NUB1 to induce ERα-independent cell cycle arrest in ER-negative BC cells. Further, we proposed that NUB1 drives cell cycle arrest in BC, with ER status determining its differential effects and predictive value between ER+ and ER− subtypes. In vivo, cytoplasmic and nuclear NUB1 expression levels predict survival outcomes in FEC-treated patients, with low NUB1 correlating with poor prognosis in ER-defined subgroups.

This study aims to elucidate the mechanistic role and predictive potential of NUB1 in FEC-induced cell cycle regulation and its interplay with ERα in BC, combining in vitro and clinical investigations.

## 2. Materials and Methods

### 2.1. Ethical Approval and Tissue Specimen Collection

Primary BC patients at Hospital Universiti Sains Malaysia (HUSM) and Hospital Canselor Tunku Mukhriz (HCTM) who completed FEC treatment between 2011 and 2018 and fulfilled the inclusion and exclusion criteria were included in the study. Inclusion criteria included Malaysian females aged 18–75 years with primary BC. The exclusion criteria were other prior treatments and known hypersensitivity to FEC compounds. Archival formalin-fixed, paraffin-embedded (FFPE) samples were obtained from HCTM and HUSM, totalling 85 samples, including 51 ER-negative and 34 ER-positive cases. The samples were further classified based on tumour grade: 5 patients (Grade I), 23 patients (Grade II), and 34 patients (Grade III). The tumour stage in the remaining patients was unknown. Additionally, 90% of the patients had a positive lymph node status. The patients’ survival status was retrieved from Malaysia’s National Registration Department. The sample collection specifically adhered to the Declaration of Helsinki and the Malaysian Guidelines for Good Clinical Practice. Institutional review board approval was obtained from Universiti Sains Islam Malaysia (USIM) (USIM/JKEPI2020-114), Hospital Universiti Sains Malaysia (HUSM) (USM/JEPeM/19120957), and Universiti Kebangsaan Malaysia (UKM) (UKM PPI/111/8/JEP-2020-042).

### 2.2. Cell Lines and FEC Treatment

Cancer cell lines (MCF-7 and MDA-MB-231) were acquired from ATCC (Manassas, VA, USA) and cultured in RPMI 1640 medium supplemented with 10% foetal bovine serum (FBS). MDA-MB-231 (ER-negative) and MCF-7 (ER-positive) BC cell lines were selected to investigate the role of NUB1 in modulating the response to FEC chemotherapy, based on ER status. These cell lines represent distinct BC subtypes with differing clinical behaviours and treatment sensitivities. The mycoplasma-free cells were stored in liquid nitrogen. FEC from Sigma-Aldrich was stored at −20 °C in DMSO. FEC concentrations (low, medium, and high) were prepared at a 22:1:11 molar ratio using RPMI media. Cells were maintained in RPMI 1640 (Thermo Scientific, Waltham, MA, USA) with 10% FBS at 37 °C with 5% CO_2_ and passaged every six to seven days. Seeded on P60 plates, cells were treated with FEC in triplicate at a 22:1:11 molar ratio (Low FEC 0.7 µM, 0.03 µM, 0.35 µM/Medium FEC 1.1 µM, 0.1 µM, 0.5 µM/High FEC 2.2 µM, 0.2 µM, 1.1 µM) for 24 and 48 h. The clinical dosage followed the FNCLCC PACS 01 trial recommendations [13]. In vitro FEC treatment used a 22:1:11 molar ratio, testing three dosages, as mentioned earlier, and two durations (24 and 48 h) for BC prevention in vitro.

### 2.3. Cell Cycle Analysis Using Flow Cytometry

The cells were treated with three FEC concentrations (low, medium, and high) in triplicate for 24 and 48 h. After treatment, the cells were harvested by aspirating the medium, washed with PBS, and trypsinised. Detached cells were transferred to sterilised tubes, centrifuged, and fixed in 70% ice-cold ethanol. After vortexing and washing with PBS, cells were resuspended in 0.25 mL PBS, mixed with RNAse A, and incubated at 37 °C. Propidium iodide (PI) staining was performed for 30 min at room temperature. Flow cytometry analysis (Novocyte^®^ Flow Cytometer, ACEA Biosciences, San Diego, CA, USA) was performed on the stained cells to assess the cell cycle.

### 2.4. Western Blot

Cells were treated and incubated as previously described, then washed with ice-cold PBS and lysed with a buffer containing 100× protease inhibitor (Merck, Darmstadt, Germany) and 10× RIPA lysis buffer. Total protein was extracted, quantified, and mixed with Laemmli sample buffer for SDS-PAGE. The samples were heated, loaded onto gel wells, and resolved by SDS-PAGE (8%). Proteins were transferred to a PVDF membrane, blocked with BSA, and incubated overnight with NUB1 and β-actin antibodies. HRP-conjugated secondary antibody was used, and the ECL substrate was used to visualise the bands. The ImageJ software was used to determine the relative protein densities. The experiments were performed in triplicate.

### 2.5. Micro-Sectioning of Paraffin-Embedded Tissue Sections

Formalin-fixed, paraffin-embedded (FFPE) BC tissue blocks obtained from HCTM and HUSM were placed on a cooling plate for 10 min before being sectioned. Microtome-cut 3 µm tissue ribbons were then transferred to a warm water bath, and tissue sections were placed on slides (25 mm × 75 mm; Thermo Fisher Scientific, Waltham, MA, USA). The slides were air-dried and labelled for further analysis.

### 2.6. Immunostaining of Paraffin-Embedded Tissue Sections

Slides were heated at 60 °C for 10 min for wax melting and subsequently underwent deparaffinisation, rehydration, and antigen retrieval. High-pressure antigen retrieval was conducted in a decloaking chamber using a DAKO buffer. After cooling in PBS, the staining was continued with reagent preparation. Primary NUB1 antibody incubation for two hours initiated the staining run, which was performed with an auto-stainer (Agilent DAKO, Link 48) and an ultraview diaminobenzidine (DAB) kit (Agilent DAKO, Denmark). Brown staining indicates immunoreactivity. Two pathologists independently scored NUB1 expression in BC tissues. Using a median cutoff (5.00), expression levels were categorised as high (IPS ≥ 5.00) or low (IPS < 5.00), with high interobserver concordance observed between the independent assessments. Nuclear and cytoplasmic staining of the tumour cells was recorded separately. The intensity percentage score (IPS) was calculated by multiplying the percentage of positive cells by staining intensity. The formula used for IPS is P × I, with a maximum score of 300 [26]. Non-neoplastic tonsil tissues served as a reference control to comparatively assess NUB1 expression levels in BC tissues.

### 2.7. Statistical Analysis

Statistical analyses were performed using SPSS (version 15.0; Chicago, IL, USA) and GraphPad Prism version 9.2.0. Cell cycle analysis results are expressed as the mean ± standard deviation (SD). The means of the experimental groups were compared using a paired *t*-test. Kaplan–Meier curves and log-rank tests were used to analyse the survival rate with NUB1 levels. The chi-square test was used to analyse BC clinicopathological factors based on ER groups. The general linear model (GLM) multivariate analysis was used to analyse the effects of prognostic factors on the NUB1 mean IPS. The Cox regression hazards model was used to determine the individual hazard ratios in BC patients. Statistical significance was set at *p* < 0.05.

## 3. Results

### 3.1. Anti-Proliferative Effects of FEC Treatment on BC Cells Through the Expression of Cell Cycle Regulatory Proteins

**Figure 1:**
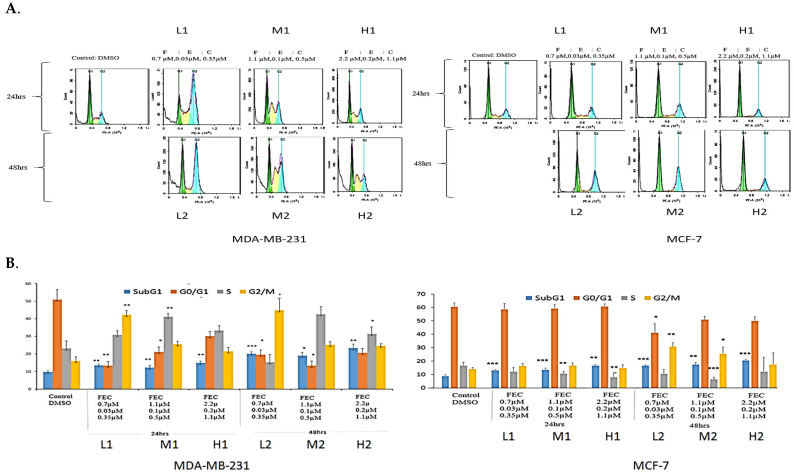
The effects of FEC treatment on cell cycle profiles of MDA-MB-231 and MCF-7 cell lines. (**A**) MDA-MB-231 and MCF-7 cell lines were subjected to three concentrations of FEC (Low (L), Medium (M), and High (H)), with treatment groups incubated for 24 h (L1, M1, H1) and 48 h (L2, M2, H2). Notably, G2/M and S-phase cell cycle arrests were observed in MDA-MB-231. The MCF-7 cell line underwent G2/M phase cell cycle arrest. (**B**) Colour-coded representation of cell cycle phases: Sub-G1 (blue), G1 (orange), S (grey), and G2/M (yellow). The mean values of cell cycle phases in the treatment groups were compared with the control group using a paired *t*-test. Data were presented as the mean ± SD (*n* = 9). The mean values of the cell cycle phases of treatment groups were compared with the mean values of cell cycle phases of a control group by using a paired *t*-test; *p* < 0.05 *, *p* < 0.01 **, *p* < 0.001 ***.

The effect of FEC treatment on MDA-MB-231 cell cycle profiles was analysed using FACS (Figure 1A). Low FEC doses caused significant G2/M arrest (*p* = 0.01, *p* = 0.04), whereas FEC-Medium at 24 h and FEC at 48 h induced significant S-phase arrest (*p* = 0.01, 0.02) (Figure 1B). FEC-Medium-48 h and FEC-High-24 h showed increased S-phase cells, though the difference was not statistically significant (Figure 1A).

The MCF-7 cell cycle showed G2/M arrest at FEC-Low-48 h and FEC-Med-48 h (*p* = 0.01 and 0.04, respectively). No S-phase arrest was observed in MCF-7 cells (Figure 1B). However, a significant decrease in the percentage of ER-positive MCF-7 cells in S-phase was noted (Figure 1B). Statistical paired *t*-test analysis confirmed these findings (Figure 1B). The sub-G1 population indicated DNA fragmentation, a sign of apoptosis or cell death [27]. All treatment groups displayed significant cell death in the sub-G1 phase in both cell lines. Higher cell death at the Sub-G1, in a dose- and time-dependent manner, indicated the effect of FEC compared to the control group. The results highlighted the anti-proliferative effects of FEC on BC cell lines by altering ERα in MCF-7 cells and upregulating NUB1 expression in the cell cycle of both cell lines.

### 3.2. FEC Upregulated NUB1 and Downregulated ERα Expression

**Figure 2:**
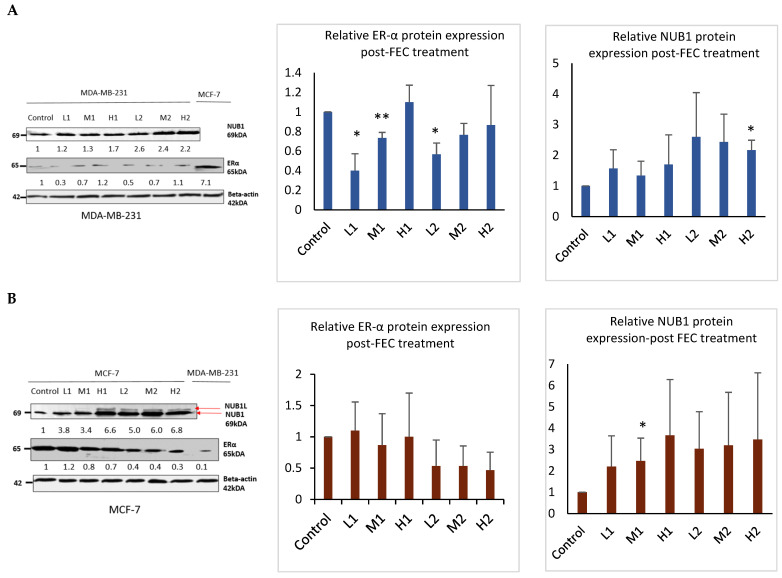
NUB1 and ER-α protein expression in MDA-MB-231 and MCF-7 after FEC treatment. (**A**,**B**) Post-FEC treatment, NUB1 and ER-α protein expression levels were assessed in MDA-MB-231 and MCF-7 cells. Relative protein intensities were quantified using densitometry analysis. This figure illustrates the changes in NUB1 and ER-α protein expression in response to FEC treatment in MDA-MB-231 and MCF-7 cell lines. Bars show the mean band intensity from three biological replicate western blots, normalised to β-actin. Wider error bars may stem from biological heterogeneity in FEC treatment response. Densitometry measurements provide a quantitative assessment of the relative protein intensities, enhancing the accuracy of the observed alterations. *p* < 0.05 *, *p* < 0.01 **.

Western blotting analysis confirmed that FEC treatment upregulated NUB1 in both cell lines and decreased ERα protein expression in the ER-positive BC cell line (Figure 2). NUB1 exhibited dose- and time-dependent upregulation in MDA-MB-231 and MCF-7 cell lysates post-FEC treatment (Figure 2A,B). High FEC concentrations for 48 h induced NUB1 expression, also triggering NUBIL (NUB1 long; an isoform of NUB1) expression in MCF-7 cells (Figure 2B). Post-FEC treatment, MCF-7 lysates exhibited a dose- and time-dependent trend toward reduced ERα protein expression, most evident at high dose/48 h, although statistical significance was limited due to biological variability (Figure 2B). In contrast, MDA-MB-231 lysates displayed no functionally meaningful ERα expression (Figure 2A). MDA-MB-231 cells may express very low or trace amounts of ERα that fall below the threshold of functional relevance [28]. Nonetheless, these low levels can still be detected using highly optimised immunoblotting and a sensitive antibody, even though they are clinically insignificant. ERα downregulation in response to NUB1 induction occurs primarily in ER-positive MCF-7 cells.

These findings support a mechanistic model in which FEC-induced NUB1 upregulation downregulates ERα predominantly in ER-positive MCF-7 cells, contributing to reduced S-phase population and G2/M cell cycle arrest. In MDA-MB-231 cells, the disruption of DNA synthesis in the S-phase of MDA-MB-231 cells appears to be ERα-independent.

### 3.3. NUB1 Expression Patterns and Subcellular Localisation in BC

**Figure 3:**
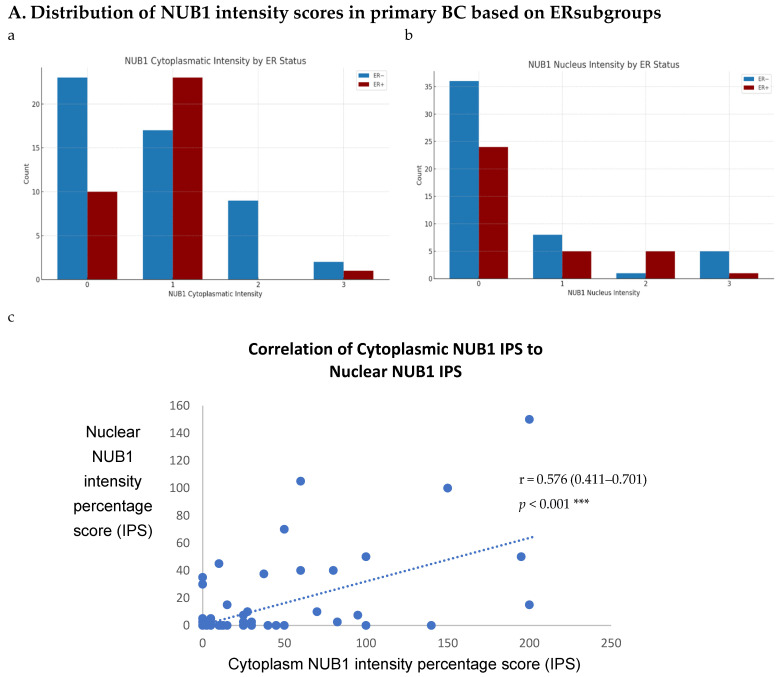

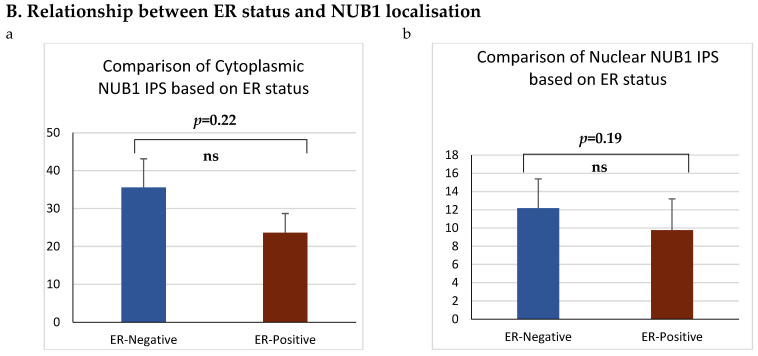

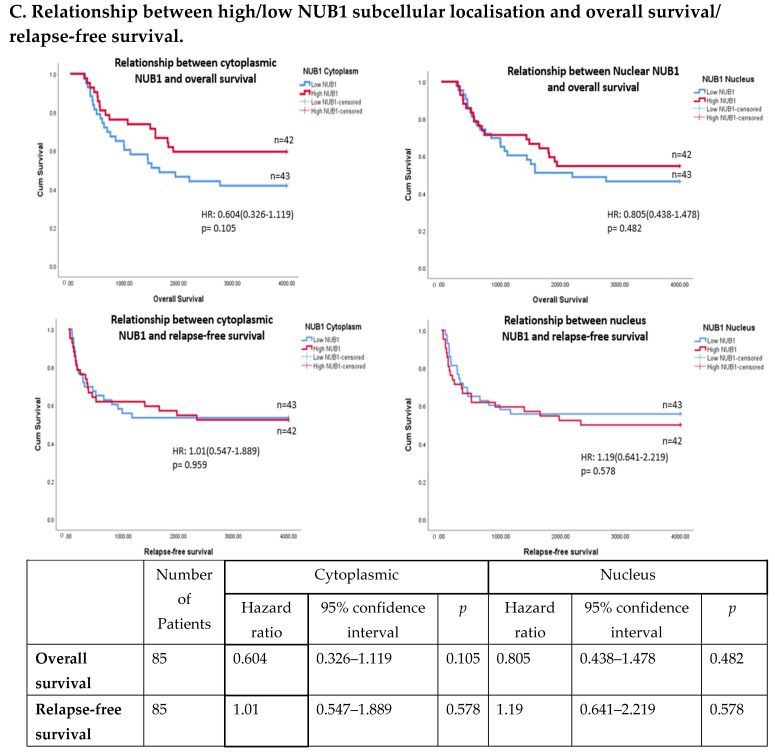

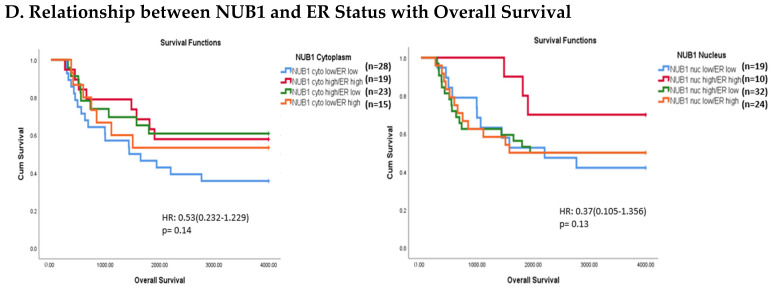

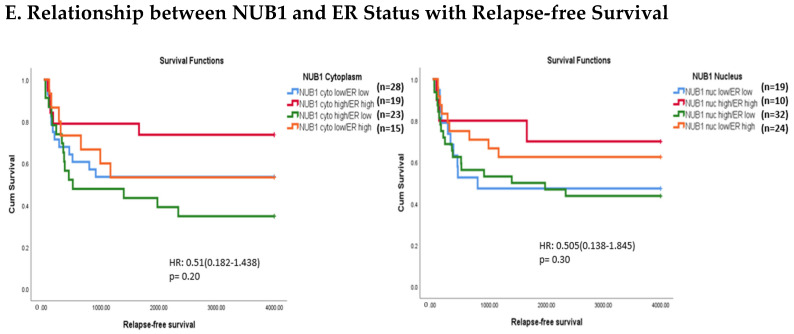
(**A**). Distribution of NUB1 immunohistochemical staining scores in primary BC. (a) NUB1 cytoplasmic staining scores in the ER− and ER+ subgroup; (b) NUB1 nuclear staining scores in the ER− and ER+ subgroup; and (c) correlation of NUB1 cytoplasmic IPS with nuclear IPS, R = 0.576. (**B**). Relationship between ER status and NUB1 localisation. Bar charts according to ER status and the subcellular localisation of NUB1 measured as an IPS (ns = non-significant). (a) Patient numbers are shown in the distribution of cytoplasmic NUB1 IPS and medians according to ER-negative and ER-positive breast cancers. (b) Nuclear NUB1 IPS and medians according to ER status and the number of patients. *p* values indicate significance. (**C**). Relationship between high/low NUB1 subcellular localisation and overall survival/relapse-free survival. Overall survival curves were plotted according to NUB1 status using Kaplan–Meier curves and log-rank test. The figure legends explicitly state confidence intervals (95% CI) for hazard ratios. (**D**). Relationship between NUB1 and ER status with overall survival for prognosis in BC patients using the log-rank test. Significant *p* values and hazard ratios are indicated in graphs by using Cox regression hazard models. (**E**). Relationship between NUB1 and ER status with relapse-free survival for prognosis in BC patients using the log-rank test. *p* values and hazard ratios are indicated in graphs by using Cox regression hazard models.

To explore NUB1’s impact on BC progression, we analysed its expression patterns in a sizable cohort of primary breast tissues and examined OS and relapse-free survival (RFS) outcomes and clinicopathological parameters. Immunohistochemistry (IHC) was performed on 85 human BC tissues, categorised into two groups: 51 ER-negative and 34 ER-positive cases, to provide insights into NUB1 subcellular localisation. IHC revealed cytoplasmic and/or nuclear NUB1 localisation, with a higher NUB1 expression in ER-negative patients (3+ score) than in ER-positive patients (Figure 3A). Of the 51 ER-negative cases, 31 were NUB1 positive, and out of 34 ER-positive cases, 24 were NUB1 positive. Comparatively, higher expression of NUB1, with a higher 3+ score, was observed in the ER-negative group than in the ER-positive group (Figure 3A). Cytoplasmic staining was found in 61% of the tumours, nuclear staining in 29%, and both in 16%.

ER status was examined with clinicopathological criteria, revealing a significant association between the nuclear and cytoplasmic NUB1 IPS. We observed a strong, statistically significant relationship between the nuclear NUB1 IPS and cytoplasmic NUB1 IPS (r = 0.57 (0.41–0.70), *p* = 0.001) (Figure 3A). Cytoplasmic NUB1 levels were significantly higher in ER-negative tumours (mean IPS: 35.5) compared to ER-positive BCs (mean IPS: 23.6) (Figure 3B).

### 3.4. Multivariate Analysis: NUB1 Expression Association with Survival in BC

The expression of NUB1 was evaluated by immunohistochemical staining and was correlated with overall patient survival, as determined by a 10-year follow-up. Survival analysis revealed that lower cytoplasmic and nuclear NUB1 expression was associated with poorer OS (HR = 0.61; 95% CI = 0.32–1.11, with non-significant *p* = 0.10). High NUB1 expression failed to improve RFS (Figure 3C). Subgroup analysis based on ER status indicated that high NUB1 and ER expression led to better OS and RFS at both cytoplasmic (*n* = 19) and nuclear (*n* = 10) levels with low hazard ratios (0.53 and 0.37, respectively) and non-significant *p* values (0.14 and 0.13, respectively) (Figure 3D). The same subgroup with high NUB1 and high ER also seemed to have better RFS at both cytoplasmic (*n* = 19) and nuclear (*n* = 10) levels with low hazard ratios (0.51 and 0.50, respectively) and non-significant *p* values (0.20 and 0.30, respectively) (Figure 3E). These findings (Figure 3D,E) suggest that the concurrent depletion of both NUB1 and ER is associated with reduced OS and an elevated relapse rate in patients with BC.

Conversely, depletion of NUB1 in conjunction with low ER levels appeared to exacerbate recurrence in BC patients, while higher NUB1 expression coupled with high ER levels may serve as a protective factor against recurrence (Figure 3E). We found no significant clinicopathological variables according to the ER status in patients with BC (Table 1). In a multivariate analysis that integrated clinical findings with gross and microscopic observations and data on BC tissues, we explored the interconnection between clinicopathological characteristics and IPS (cytoplasmic and nuclear). No significant association emerged between the correlations of predictive factors and NUB1 mean IPS, as presented in Table 2.

## 4. Discussion

BC progression is a multifaceted process driven by oncogenic activation and tumour suppressor inactivation, critically influencing patient survival and therapeutic response [2,3]. While oncogenic pathways like HER2 and cyclin D1/CDK4 have been extensively characterised in BC [29,30], the roles of tumour suppressor genes, particularly those modulating chemotherapy response, remain understudied. Our study identifies NUB1 as a novel tumour suppressor and predictive biomarker in BC, with dual roles in FEC chemotherapy response and ERα regulation. Unlike proliferation markers such as Ki-67, which broadly predict tumour aggressiveness [31], NUB1’s role in FEC-specific cell cycle arrest offers a chemotherapy-focused prognostic tool. Similarly, while CDK4/6 inhibitors target ER+ BC [32], NUB1’s ER-independent mechanism in ER− subtypes may address unmet needs in triple-negative BC management.

NUB1 is characterised initially as a negative regulator of NEDD8 conjugation [22]. NUB1 exhibits conflicting roles across cancers: it suppresses gastric cancer via p27^Kip1^ upregulation [24], yet paradoxically induces cell cycle arrest in BC [25]. NUB1’s tissue-specific role underscores the complexity of its function, which our data reveal is further modulated by ER status. Our study demonstrates that FEC chemotherapy upregulates NUB1 expression in BC cells, inducing cell cycle arrest and suppressing proliferation, while concurrently downregulating ERα in ER-positive cells. The DNA damage caused by FEC components, particularly epirubicin-mediated topoisomerase II inhibition [11] and cyclophosphamide-induced DNA crosslinking [33], triggers interferon signalling pathways [18] that are known to upregulate NUB1 expression [23]. This aligns with our observations of dose- and time-dependent NUB1 upregulation in both ER-negative MDA-MB-231 cells (showing significant S-phase (24 h: *p* = 0.04; 48 h: *p* = 0.02) and G2/M-phase arrest (24 h: *p* = 0.01; 48 h: *p* = 0.02) and ER-positive MCF-7 cells (exhibiting G2/M arrest (48 h: *p* = 0.01).

The NUB1-mediated cell cycle regulation appears to operate through a dual mechanism: first, by stabilising p27^Kip1^ [24], and second, by downregulating ERα, which we observed post-FEC treatment in ER+ MCF-7 cells. This ERα reduction likely contributes to decreased CDK4/6 and cyclin D1 expression [29], inducing a synergistic cell cycle arrest [34]. Our findings are particularly significant in ER-negative cells, which appear to rely more heavily on this NUB1-p27^Kip1^ axis for cell cycle control in the absence of functional ERα signalling. The interferon-NUB1-p27^Kip1^-ERα pathway we propose provides a plausible mechanism for FEC’s differential effects in ER+ versus ER− BC cell lines, warranting further investigation into this pathway’s therapeutic potential.

The inverse relationship between NUB1 and ERα post-FEC treatment suggests a compensatory mechanism where NUB1-mediated regulation may offset ERα loss in ER+ cells. ER-negative cells, lacking ER hormonal signalling, may rely more heavily on NUB1 to enforce cell cycle checkpoints. This aligns with studies showing ERα regulates cyclin D1 and CDK4/6 in MCF-7 cells [34,35], whereas ER-negative cells depend on alternative pathways, such as p27^Kip1^ modulation [36]. Our observation that FEC failed to induce S-phase arrest in ER-positive cells further underscores ERα’s role in mediating chemoresistance, possibly through cyclin A/CDK2 activation [37,38].

Clinically, low cytoplasmic NUB1 expression correlated with poorer overall survival (OS) in ER-negative patients (HR = 0.60; 95% CI = 0.32–1.11, *p* = 0.10), while low nuclear NUB1 predicted worse OS in ER-positive subgroups. Although statistically non-significant, these trends mirror findings by Tan et al. (2024), who linked low NUB1 to poorer OS in BC [25]. Notably, high cytoplasmic NUB1 in ER-positive patients improved relapse-free survival (RFS) (HR = 0.51; *p* = 0.20), suggesting a protective role contingent on ER signalling. Conversely, ER-negative patients with low nuclear NUB1 exhibited heightened recurrence risk, emphasising NUB1’s divergent predictive potential across subtypes. These results uniquely highlight ER status as a critical modifier of NUB1’s clinical relevance. Another recent study complements that low NUB1 triggers tumour growth in hepatocellular carcinoma (HCC) via the PCNA-NEDD8 pathway, a well-established mechanism linked to elevated proliferation [39].

The study’s limitations temper interpretation. First, the small cohort (*n* = 85) likely underpowered the subgroup analyses, as evidenced by wide confidence intervals (e.g., 95% CI = 0.32–1.11 for OS). An a priori power calculation, which was not conducted here, is essential in future studies to detect meaningful clinical effects. Our post-hoc power analysis of the study indicated the need for a larger sample size of 217 patients to achieve statistical significance. Second, manual NUB1 scoring introduced variability; automated platforms could mitigate this bias. While our study relied on two pathologists to reduce manual scoring variability, observer-dependent discrepancies remain a concern.

Despite these constraints, our findings propose actionable insights. Mechanistically, NUB1’s upregulation post-FEC suggests it may counteract ERα loss in ER-positive cells, potentially inducing cell cycle arrest and delaying resistance. Clinically, NUB1’s predictive stratification by ER status could refine FEC therapy selection. For example, ER-negative patients with low cytoplasmic NUB1 (mean IPS = 1.1% vs. 60.0%) in high-cytoplasmic NUB1 tumours may benefit from alternative regimens, such as CDK4/6 inhibitors, to bypass defective cell cycle checkpoints [40]. Future studies should validate NUB1 in larger, multi-centre cohorts and integrate multi-omics approaches (e.g., RNA sequencing) to resolve its interaction with ERα and chemoresistance pathways. Additionally, preclinical models could test NUB1 modulators (e.g., IFN-γ inducers) to enhance FEC efficacy [23].

## 5. Conclusions

This study integrates in vitro and clinical data to highlight NUB1 as a potential predictive biomarker in FEC-treated BC; however, its clinical utility remains exploratory, pending prospective validation in larger, multicentre cohorts. Future trials stratifying patients by ER/NUB1 profiles are imperative to optimise FEC therapy and enable personalised strategies for high-risk subtypes.

## 6. Patent

Our study builds on a patented IHC methodology (Malaysian Patent Application No. PI2024002978), titled “Method for Detecting the Expression of NUB1 in Human Tumour Tissue”, filed on 1 May 2024, and developed as part of this research study.

## Figures and Tables

**Table 1:** Clinicopathological variables according to ER status in BC patients.

Clinicopathological Factors	ER-Negative (*n* = 51)	ER-Positive (*n* = 34)	Overall (*n* = 85)	*p*-Value
HER 2 Receptor Status				
Negative	16 (55.17%)	13 (44.82%)	29	0.354
Positive	22 (66.66%)	11 (33.33%)	33	
Node Status				
Negative	6 (75%)	2 (25%)	8	0.348
Positive	44 (57.89%)	32 (42.10%)	76	
Histologic Grade				
Grade I	4 (80%)	1 (20%)	5	0.814
Grade II	15 (65.21%)	8 (34.78%)	23	
Grade III	23 (67.64%)	11(32.34%)	34	
Age				
<50 years	22 (57.89%)	16 (42.11%)	38	0.824
≥50 years	29 (61.7%)	18 (38.29%)	47	
Median(Range)	52 (32–73)	51(27–70)	52 (27–73)	
Tumour Size				
≤2 cm	12 (70.58%)	5 (29.14%)	17	
˃2 cm	38 (56.71%)	29 (43.28%)	67	
Median(Range)	4 (0.5–11.3)	5 (1–16)	4 (0.5–16)	0.298

**Table 2:** Summary of multivariate analysis of prognostic factors with NUB1 mean IPS.

Variable	Events 85	Cytoplasmic			Nuclear		
		Mean IPS	95% Confidence Interval	*p*	Mean IPS	95% Confidence Interval	*p*
Age, year							
≥50	47	28.9	21.470–52.896	0.780	9.30	−2.592–15.505	0.496
<50	38	31.8	6.815–42.827		13.4	−7.648–15.505	
Size, cm							
≤2	17	41.9	20.469–72.656	0.144	17.7	−3.338–30.213	0.096
>2	67	24.7	13.025–39.834		7.3	−5.157–12.078	
Lymph nodes							
Positive	76	29.8	13.979–40.351	0.652	12.1	−1.994–14.960	0.383
Negative	8	37.8	21.361–79.055		3.1	−14.379–22.713	
Grade							
1	5	88.0	−43.802–103.802		40.0	−44.948–49.948	
2	23	21.4	8.866–46.865	0.259	0.97	−10.484–13.945	0.214
3	34	32.8	18.793–50.398		8.30	−0.625–19.694	
Histology							
Ductal	75	31.9	18.417–43.395	0.280	11	−1.348–14.711	0.896
Lobular	5	28.5	−7.186–97.186		0	−33.551–33.551	
Other	5	7.0	−48.802–98.802		0	−47.448–47.448	
ER							
Negative	51	35.0	24.520–55.966	0.248	12.4	−0.917–14.105	0.595
Positive	34	23.0	2.647–39.674		9.1	−9.700–14.105	
HER2							
Negative	29	31.6	13.026–47.130	0.571	8.8	−5.025–16.900	0.927
Positive	33	25.6	16.386–50.164		9.3	−4.719–16.997	
Menopausal status							
Pre	29	34.9	9.578–50.573	0.513	14.0	−8.405–17.951	0.487
Post	56	27.8	17.663–47.291		9.6	−2.795–16.253	

Significant *p* values (*p* ≤ 0.05); Pearson correlation analysis. NUB1 mean IPS, 95% confidence intervals; multivariate analysis.

## Data Availability

All data have been incorporated into the article.

## Abbreviations

The following abbreviations have used in this manuscript:

ATCC	American Type Culture Collection
BC	Breast Cancer
CDK	Cyclin-Dependent Kinase
CI	Confidence Interval
DAB	Diaminobenzidine
DMSO	Dimethyl Sulfoxide
ECL	Enhanced Chemiluminescence
Erα	Oestrogen Receptor alpha
FACS	Fluorescence-Activated Cell Sorting
FBS	Fetal Bovine Serum
FEC	5-Fluorouracil, Epirubicin, Cyclophosphamide (chemotherapy regimen)
FFPE	Formalin-Fixed Paraffin-Embedded
FRGS	Fundamental Research Grant Scheme
GLM	General Linear Model
HER2	Human Epidermal Growth Factor Receptor 2
HR	Hazard Ratio
HRP	Horseradish Peroxidase
IHC	Immunohistochemistry
IPS	Intensity Percentage Score
NEDD8	Neural precursor cell-expressed developmentally downregulated protein
NUB1	NEDD8 Ultimate Buster 1
OS	Overall Survival
PBS	Phosphate-Buffered Saline
PCNA	Proliferating Cell Nuclear Antigen
PI	Propidium Iodide
PR	Progesterone Receptor
PVDF	Polyvinylidene Difluoride
RFS	Relapse-Free Survival
RIPA	Radioimmunoprecipitation Assay Buffer
RPMI	Roswell Park Memorial Institute medium
SCF	Skp, Cullin, F-box containing complex (ubiquitin ligase)
SDS-PAGE	Sodium Dodecyl Sulfate-Polyacrylamide Gel Electrophoresis
TME	Tumour Microenvironment
